# Unlocking the potential of bone marrow in postmortem forensic toxicology: current evidence and future perspectives

**DOI:** 10.1093/jat/bkag014

**Published:** 2026-02-20

**Authors:** Sofia Vanerio, Nicola Galante, Alessandro Ravelli, Roberta F Bergamaschi, Alessio Battistini, Sara Casati

**Affiliations:** Laboratory of Forensic Toxicology, Department of Biomedical, Surgical and Dental Science, University of Milan, Via Luigi Mangiagalli, 37, Milan, 20133, Italy; Department of Biomedical Sciences for Health, Via Luigi Mangiagalli, 37, Milan, 20133, Italy; Laboratory of Forensic Toxicology, Department of Biomedical, Surgical and Dental Science, University of Milan, Via Luigi Mangiagalli, 37, Milan, 20133, Italy; Laboratory of Forensic Toxicology, Department of Biomedical, Surgical and Dental Science, University of Milan, Via Luigi Mangiagalli, 37, Milan, 20133, Italy; Laboratory of Forensic Toxicology, Department of Biomedical, Surgical and Dental Science, University of Milan, Via Luigi Mangiagalli, 37, Milan, 20133, Italy; Laboratory of Forensic Toxicology, Department of Biomedical, Surgical and Dental Science, University of Milan, Via Luigi Mangiagalli, 37, Milan, 20133, Italy; Fondazione IRCCS Ca’ Granda Ospedale Maggiore Policlinico, Via Francesco Sforza, 35, Milan, 20135, Italy

## Abstract

Bone marrow (BM) has emerged as a valuable alternative matrix in postmortem toxicology, when conventional samples such as blood or soft tissues are unavailable, degraded or contaminated. BM, due to its protecting anatomical site within the medullary cavity, slows down decomposition, limits environmental and microbial interference and enables the preservation of xenobiotics over extended postmortem intervals. This review summarizes the updated literature on BM anatomy, sampling site, xenobiotic distribution and stability, as well as the influence of postmortem redistribution (PMR) and putrefaction for toxicological interpretation of the analytical results. Evidence indicates that drug distribution in BM is governed by tissue vascularity and analyte physicochemical properties, with lipophilic compounds often reaching higher concentrations in BM than in blood. Numerous studies demonstrate the long-term stability of drugs such as amphetamines, benzodiazepines, sedative-hypnotics and ethanol in BM, even in severely decomposed, burned, or skeletonized remains. While BM shows reduced susceptibility to PMR and putrefaction compared to blood and soft tissues, post-collection stability depends on proper storage conditions. Findings from human forensic cases assess the detectability of a wide range of illicit and therapeutic substances in BM, supporting its utility in reconstructing ante-mortem drug exposure when traditional matrices are compromised. Despite promising results, data remain limited, and further research is needed to refine interpretative frameworks and establish standardized protocols for BM sampling, analysis, and toxicological evaluation.

Highlights

- BM as an emerging valuable alternative matrix in postmortem toxicology when blood is unavailable.
- BM anatomy, sampling, xenobiotic distribution, drug stability and the influence of postmortem redistribution (PMR) or decomposition on analytical results have been reviewed.
- Findings support the utility of BM in reconstructing ante-mortem drug and ethanol exposure when traditional matrices are compromised.

## Introduction

In postmortem toxicology, several biological matrices are commonly analyzed for toxics, illegal and therapeutical drugs as well as their metabolites. Conventional matrices collected during the autopsy include peripheral and cardiac blood, urine, bile, vitreous humor, gastric content, hair and visceral tissues (i.e. brain, lungs, liver, kidney, spleen, muscle). However, these matrices may be limited, or totally unavailable and unsuitable for analysis in cases involving severely decomposed, skeletonized, bloodless or embalmed bodies [1]. Advances in analytical techniques have expanded the forensic applicability of non-traditional matrices such as skeletal and adipose tissues, teeth, nails, and bone ­marrow (BM) [2]. BM is a connective tissue located within the medullary cavity of bones, and it has emerged as a promising alternative matrix, when conventional samples are compromised by decomposition, trauma, or environmental exposure (e.g. fire) [3]. Despite its vascular nature, BM decomposes more slowly than other tissues due to its anatomical site, since it consists in a natural barrier against external contaminants and putrefaction [4]. However, this protection is contingent on the structural integrity of the surrounding bone. Indeed, in cases of bone degradation, contaminants and microorganisms infect the exposed marrow, reducing its reliability for toxicological analysis [5].

Considering the high potential of this promising biological matrix for forensic toxicological analyses, a literature search was conducted on PubMed using the terms “bone marrow” in combination with “toxicology,” “forensic,” “postmortem,” and “analysis.” References from retrieved articles were also cross-checked. Only studies written in the English language and published between January 1978 and December 2025 (47 years) were included. This narrative review summarizes the updated literature on BM anatomy, sampling site, xenobiotic distribution and stability, and the influence of postmortem redistribution (PMR) or putrefaction for toxicological interpretation of the analytical results.

## Anatomy, histology and function of bone marrow

Anatomically, BM is located within the central cavities of long bones and axial skeleton. It consists of a complex and highly organized vascular connective tissue composed of hematopoietic cells at various stages of development, adipocytes, and reticuloendothelial cells, all supported by a trabecular framework [6]. BM has an extensive vascular network, supplied by nutrient arteries that enter the bone through canals and branch into arterioles and capillaries. Blood flow follows a circular pattern, moving from the central longitudinal vein to the venous sinuses and then back toward the center. Unlike many other tissues, BM lacks lymphatic drainage [7]. BM is classified into two types: red (hematopoietic) and yellow (fatty), based on the predominance of cellular components. With age, red marrow gradually converts into yellow marrow. In a middle-aged adult, red marrow contains approximately 60% hematopoietic cells, while yellow marrow consists of about 95% adipocytes. Red marrow, also known as myeloid tissue, includes stromal connective tissue that provides structural support for hematopoietic cells, white blood cells, macrophages, and a dense capillary network [8]. The distribution of red and yellow marrow varies along with anatomical sites: rib and iliac crest marrow are predominantly hematopoietic, while femoral marrow is primarily fatty [9]. Histologically, BM consists of a cluster of hematopoietic cells and adipocytes surrounded by vascular sinuses. In the hematoxylin and eosin-stained section, mature erythroid and myeloid cells, adipocytes, mast cells and megakaryocytes are identifiable, while immature progenitor cells and lymphoid cells are harder to distinguish [7]. As the primary hematopoietic organ, BM is the site for maturation of erythrocytes, granulocytes, monocytes, lymphocytes, and platelets. This function is supported by a specialized microenvironment consisting of reticular cells, endothelial cells, macrophages, adipocytes and extracellular matrix components, which collectively provide cytokines and growth factors necessary for hematopoietic cell proliferation, differentiation and maturation [7, 10].

## Bone marrow sampling

Currently, no standardized protocols exist for the collection of postmortem BM. BM can be obtained from various anatomical sites, including (i) femur (by cutting through cortical bone), (ii) ribs (by squeezing and collecting the dense tissue) or (iii) vertebral bodies (through trocar aspiration) [11].

BM collection from the femur typically involves traverse sectioning of the bone at the superior third, either proximally or medially [12]. Once the cortical layer is cut or fractured, the medullary cavity becomes accessible, allowing direct sampling of the fatty marrow content [13].

Ribs are an accessible source of red BM. During autopsy, after removal of the rib block by transecting the costal cartilages a few centimeters laterally to the sternum, each rib is cut approximately five centimeters from their distal end. The dark red marrow then oozes out of the trabecular bone, by compressing the ends of the rib [11]. This approach provides a practical and minimally invasive method for obtaining a representative portion of red marrow, suitable for subsequent toxicological analyses [8]. Alternatively, vertebral BM can be collected through aspiration. Using a syringe connected to a marrow needle, the lower thoracic vertebral bodies are punctured to obtain approximately 2–5 mL of liquid marrow, which is particularly rich in hematopoietic cells [14].

## Xenobiotics distribution in bone marrow and its comparison with blood

Once xenobiotics enter the bloodstream, they are distributed throughout the body, including the BM [11]. Overall, two major factors may influence this distribution: (i) the vascularity of the tissue, which determines the efficiency of drug delivery, and (ii) the solubility of the analyte in the target tissues, which reflects the interplay between its physiochemical properties, such as lipophilicity, and the composition of the biological matrix [15].

These principles were clearly demonstrated by Cartiser *et al*. [12], who examined postmortem caffeine levels in rib and femoral BM. Caffeine, a relatively hydrophilic compound, consistently showed higher and more stable concentrations in rib BM compared with femoral BM. The authors attributed this difference to the distinct characteristics of the two BMs: as mentioned above, rib marrow is predominantly red, highly vascularized, and relatively homogeneous, whereas femoral marrow is predominantly yellow, lipid-rich, and less uniform. These findings underscore the importance of the sampling site in BM toxicological analysis and suggest that rib BM may be more suitable for the investigation of hydrophilic substances.

However, although red BM is characterized by a dense network of sinusoids structures formed by discontinuous endothelial cells with an interrupted basal lamina, that allow the passage of circulating compounds, the pathophysiological mechanisms governing drugs distribution within BM remain poorly defined in the current literature [11].

However, it is important to compare drug concentrations in BM and blood, the gold standard matrix for postmortem toxicology, to assess the reliability of BM as an alternative matrix when blood is unavailable.

McIntyre *et al*. observed that antidepressants, antipsychotics, and benzodiazepines were consistently higher in femoral BM compared with peripheral blood. For example, diazepam concentration was approximately eight times higher in BM, and the levels of other benzodiazepines (BDZ) were at least 2.5 times greater than in blood [16]. In this context, BDZ demonstrate how lipophilicity affects drug distribution, favoring retention in lipid-rich tissues such as BM over blood. Diazepam, being highly lipophilic with a prolonged duration of action (∼48 hours), persists in these compartments, which may account for the observed concentration differences between the two matrices [17]. Conversely, other more hydrophilic substances such as opioids [18], ethanol [19] and meprobamate [3] have shown higher concentrations in blood than in BM.

These discrepancies likely reflect a combination of factors, including drug physicochemical properties, local tissue affinity, postmortem redistribution, and the stage of pharmacokinetics at the time of death. The latter further modulates drug distribution in BM compared with blood. According to Tominaga *et al*., lower drug concentrations in BM or pericardial fluid rather than peripheral blood typically indicate an early post-intake phase, when tissue distribution is still incomplete. In contrast, higher concentrations in these compartments may reflect later distribution stages. Variability can also arise from drugs with short half-lives, whose rapid degradation may alter the correlation between blood and BM [20]. Despite these insights, literature remains limited, and the dynamics governing how xenobiotic concentrations in BM correspond to those in blood remain poorly defined.

### Illicit and therapeutic drugs in human bone marrow

BM offers considerable promise as an alternative matrix for forensic toxicological analysis; however, the interpretation of drug concentrations in this matrix is still restricted [4]. Indeed, although several animal studies have been reported, research on human cadavers remains scarce, and many of these studies focus more on skeletal tissue rather than exclusively on BM. Moreover, since the extent of decomposition is a critical factor, further studies specifically addressing different post-mortem stages are necessary to better understand the relationship between substance concentrations in blood and BM.

The first documented study on drug detection in human BM was conducted by Noguchi *et al*. (1978). The researchers collected marrow samples from human skeletal remains, specifically from eight vertebrae beginning with the atlas and proceeding downward. In these samples, amitriptyline was detected at a concentration of 1.35 μg/g, demonstrating that the detection and quantitation of certain drugs in BM is feasible. It should be noted, however, that no comparison with blood concentrations was performed, as blood was unavailable due to the condition of the remains [21].

In 1986, Kojima *et al*. made a notable contribution by analyzing the BM of a thighbone from a methamphetamine user who had been strangled and buried for 5 years. Despite the prolonged postmortem interval and advanced decomposition, they successfully detected methamphetamine at a concentration of 1 mol/100 g and its primary metabolite, amphetamine, at 0.1 μmol/100 g in the BM. Based on these findings and previously established pharmacological data, the authors inferred that the individual was likely in a moderate state of methamphetamine intoxication at the time of death, possibly exhibiting drug-induced mental disturbances [22].

In 1989, Bal *et al*. identified acetaminophen (0.5 pg/g) and dextropropoxyphene (1.2 pg/g) in BM samples collected from the back of the legs and the buttocks of skeletal remains found in a forest. The remains were attributed to a man who had disappeared 20 months earlier, after leaving a suicide note in his car. The detection of these drugs in the BM suggested that the individual has ingested them before death, reinforcing the hypothesis of suicide [23].

The role of BM in acute poisoning cases was further investigated in 1992, when a study evaluated colchicine toxicity in both survivors and post-mortem cases in different matrices. While survivors BM were not analyzed, results from post-mortem cases showed a significant accumulation of colchicine in the BM, exceeding 600 ng/g, suggesting that this matrix could be valuable for ­confirming fatal poisonings [24].

Moreover, in 1997 forensic interest in BM grew with studies on bromisovalum, a sedative-hypnotic drug [25]. Maeda *et al*. analyzed skeletal remains recovered from a forested slope and detected bromisovalum in both femora, reporting concentrations of 139.7 μg/g in the left femoral BM and 36.4 μg/g in the right ­femoral BM. When compared to reported lethal blood levels (44–114 μg/mL), the higher concentration in BM suggested that bromisovalum may have contributed to the cause of death.

In another case, forensic toxicologists analyzed the remains of two victims found buried for 4 years. Triazolam was detected in two femoral BM samples and in a decomposed muscle fragment. Its concentration in the BM were similar in both victims (0.36 and 0.37 ng/g). These findings were consistent with confessions from suspects, who admitted to sedating the victims with triazolam before burying them alive [26].

McIntyre *et al*. found significant correlations between blood and femoral BM for diazepam (*r *= 0.68, *P *< .05; 9 cases) and temazepam (*r *= 0.85, *P *< .05; 10 cases), but not for nordiazepam (*r *= 0.18, *P *> .05; 7 cases). Their findings also indicated that BM concentrations for BDZ and other substances are often higher than in blood, with diazepam concentrations being about eight times greater than in blood [16].

In 2001, morphine was identified at 195 ng/g in femoral BM of a deceased opioid user. However, 6-acetylmorphine (6-AM), the primary heroin metabolite, was absent, highlighting potential issues related to metabolite stability in BM [27].

In 2011, a study investigated caffeine levels in BM. Femoral BM concentrations ranged from 51 to 6171 ng/g, while rib BM concentrations varied between 66 and 7280 ng/g. The higher correlation between rib BM and blood, in comparison of femoral BM and blood, suggested that the BM sampling site may impact the toxicological results [12].

Furthermore, in 2013, a large-scale study analyzed meprobamate in femoral blood from 99 forensic cases, demonstrating its detectability in 98 femoral BM samples and quantitation in 97 cases. The lower limit of quantification (LLOQ) was 0.2 μg/g. Blood concentrations ranged from 0.4 to 464.4 μg/mL, while BM levels varied from 0.2 to 156.6 μg/g, confirming its suitability for overdose investigations. In addition, a significant linear relationship between BM and blood meprobamate concentrations was observed (*r *= 0.85, *P *< 10^−15^) [3].

In another extensive study conducted in 2013, the distribution of 36 drugs were analyzed in blood, pericardial fluid and bone marrow aspirate (BMA) form thoracic vertebrae in 218 autopsy cases. The results showed that many drugs, such as methamphetamine, amphetamine and phenobarbital, were detected at higher concentrations in BM compared to blood, with BM levels typically 1.5 times higher. Other drugs, such as midazolam, propofol and thiamylal, showed variable distribution, with BMA levels sometimes markedly higher or lower than blood, reflecting individual pharmacokinetics and possible postmortem redistribution [20].

In 2019 [28], selective serotonin reuptake inhibitors (SSRIs) and serotonin-norepinephrine reuptake inhibitors (SNRIs) were investigated in BMA obtained from skeletal fragments in forensic cases, where blood samples were not available. BM samples showed measurable concentrations of venlafaxine (104 ng/mL), fluoxetine (84 ng/mL), paroxetine (3620 ng/mL), and citalopram (68 ng/mL), suggesting that BM can be a viable matrix for postmortem antidepressant detection.

Regarding opioids and their metabolites, the analysis of 22 forensic cases, selected based on positive blood screening for tramadol, morphine, fentanyl, codeine, or their metabolites, revealed that these substances can be quantified in BM collected from the clavicle. Tramadol and codeine concentrations in blood showed a linear correlation with those in bone and BM, supporting their reliability for postmortem analysis [18].

Finally, Willeman *et al*. [29] analyzed femoral BM in a series of homicidal poisonings that occurred in a nursing home. BM and hair were analyzed to detect and quantify drugs. The study revealed the presence of 8 substances among BDZ and antipsycotics. Cyamemazine concentrations in BM ranged between 229 ng/g and 681 ng/g, whether cyamemazine concentrations in blood ranged from 152 to 712 ng/mL, showing some consistency with BM findings. Other drugs detected in BM were tramadol (300 ng/g), *N*-desmethyltramadol (118 ng/g), diazepam (504 ng/g and 164 ng/g), nordiazepam (10.5 ng/g and 2.4 ng/g), midazolam (0.5 ng/g, 29.5 ng/g and 32.5 ng/g), scopolamine (1.5 ng/g and 0.5 ng/g) and amlodipine (5.0 ng/g). Tiapride concentrations in BM resulted below the quantification limit (< 1 ng/g). For these substances, no direct comparison with blood could be performed because blood was not available. An overview of the key studies on BM toxicology is summarized in Table 1.

**Table 1:** Overview of studies (1978–2022) reporting drugs and EtG concentration in BM and their comparison with blood: substance(s), type of BM, analytical assay, BM and blood concentrations, correlation.

Year	References	Substance(s)	Analytical assay	BM concentration	Blood concentration	Correlation with blood
1978	Noguchi *et al*. [21]	Amitriptyline	GC-FID GC-MS	1.35 μg/g	Not reported	Not reported
1986	Kojima *et al*. [22]	Methamphetamine, Amphetamine	GC-MS	Methamphetamine: 1.49 µg/g Amphetamine: 0.14 µg/g	Not reported	Not reported
1989	Bal *et al*. [23]	Acetaminophen, Dextropropoxyphene	GC-MS; TLC; HPLC	Acetaminphen: 0.0000005 µg/g Dextropropoxyphene: 0.000001 µg/g	Not reported	Not reported
1992	Rochdi *et al*. [24]	Colchicine	Radioimmunoassay	> 0.60 μg/g	Not reported	Not reported
1997	Maeda *et al*. [25]	Bromisovalum	LC-MS/MS	36.40–139.70 μg/g	Not reported	Not reported
1997	Kudo *et al*. [26]	Triazolam	GC-MS	0.00036–0.00037 µg/g	Not reported	Not reported
2000	McIntyre *et al*. [16]	Diazepam, Nordiazepam, Temazepam	GC–MS LC-MS	Diazepam: 0.94–2.34 µg/g Nordiazepam: 0.28–1.02 µg/g Temazepam: 0.20–4.40 µg/g	Not reported	*r* = 0.68 (diazepam) *r* = 0.18 (nordiazepam) *r* = 0.85 (temazepam)
2001	Raikos *et al*. [27]	Morphine, 6-MAM	FPIA GC-FID	Morphine: 0.20 µg/g 6-MAM: absent	Not reported	Not reported
2006	Schloegl *et al*. [32]	Ethyl glucuronide (EtG)	LC-MS/MS	0.77–9.36 μg/g	0.41–20.46 μg/mL	Blood/BM ± SD: 3.14 ± 2.61
2011	Cartiser *et al*. [12]	Caffeine	GC-MS/MS	0.05–6.17 µg/g (femoral) 0.07–7.28 µg/g (rib)	0.06–7.59 µg/mL	Blood/red BM ± SD 1.44 ± 0.41 Blood/yellow BM ± SD: 2.46 ± 0.71
2013	Bévalot *et al*. [3]	Meprobamate	GC-MS/MS	0.21–156.60 μg/g	0.41–464.40 µg/mL	*r* = 0.85
2013	Tominaga *et al*. [20]	36 drugs analyzed	GC/MS	BM levels typically 1.5× higher than blood		Methamphetamine: *R* = 0.96 Amphetamine: *R* = 0.72 Acetaminophen: *R* = 0.91 Phenobarbital: *R* = 1.00 Benzodiazepine: *R* = 0.91 Secobarbital: *R* = 0.80 Lidocaine: *R* = 0.80 Midazolam: *R* = 0.86 Propofol: *R* = 0.86 Tialamile: *R* = 0.91
2019	Snamina *et al*. [28]	SSRIs, SNRIs	MAE/UHPLC-TOF/MS	Venlafaxine: 0.10 µg/g Fluoxetine: 0.08 µg/g Paroxetine: 3.62 µg/g Citalopram: 0.07 µg/g	Not reported	Not reported
2022	Vandenbosch *et al*. [18]	Opioids (Tramadol, Morphine, Fentanyl, Codeine)	LC–MS/MS	Tramadol: 2.08 µg/g O-desmethyltramadol: 0.83 µg/g Codeine: 0.52 µg/g Morphine: 0.34 µg/g Fentanyl: 0.01 µg/g Norfentanyl: 0.001 µg/g	Tramadol: 2.73 µg/mL O-desmethyltramadol: 1.39 µg/mL Codeine: 0.15 µg/mL Morphine: 0.42 µg/mL Fentanyl: 0.005 µg/mL Norfentanyl: 0.003 µg/mL	Blood/BM: Tramadol: 1.31 O-desmethyltramadol: 1.68 Codeine: 0.29 Morphine: 1.25 Fentanyl: 0.41 Norfentanyl: 2.55
2022	Willeman *et al*. [29]	Cyamemazine, Tramadol, Diazepam, Midazolam, Scopolamine, Amlodipine	GC–MS LC–MS/MS	Cyamemazine: 0.23 µg/g and 0.68 µg/g Tramadol: 0.30 µg/g *N*-desmethyltramaol: 0.12 µg/g Diazepam: 0.16–0.50 µg/g Nordiazepam: 0.002–0.011 µg/g Midazolam: 0.0005–0.03 µg/g Scopolamine: 0.0005–0.002 µg/g Amlodipine: 0.005 µg/g	Cyamemazine: 0.15–0.71 µg/mL	Not reported

Abbreviations: GC-FID, gas-chromatography coupled with flame ionization detection; GC–MS: gas-chromatography coupled with mass spectrometry; TLC, thin-layer chromatography; HPLC, high-performance liquid ­chromatography; LC–MS, liquid chromatography coupled with mass spectrometry; FPIA, Fluorescence Polarization Immunoassay; MAE/UHPL-TOF/MS, microwave-assisted extraction combined with ultra-high-performance liquid chromatography coupled to time-of-flight mass spectrometry.

### Detection of ethanol in bone marrow samples

BM has emerged as a promising alternative matrix even for postmortem alcohol analysis, particularly in cases where blood samples are compromised by PMR, contamination or endogenous ethanol formation. Iskierka *et al.* analyzed ethanol in BMA samples collected from the iliac crest in 100 autopsy cases. Among these, 56 cases showed positive results for ethanol (> 0.1 mg/g) in both BM and femoral blood, while 41 cases resulted negative in both matrices, and 3 cases were positive in only one matrix (blood or BM). The average postmortem interval was 131 hours, and no cases of advanced putrefaction were reported. Mean ethanol concentrations were 0.68 ± 1.09 mg/mL in blood and 0.59 ± 0.96 mg/g in BM. A strong correlation was observed between ethanol concentrations in blood and BM (*r *= 0.97, *P *< .001) [19].

Accordingly, Maeda *et al.* examined ethanol concentrations in BMAs from 20 autopsy cases collected within 48 h postmortem. Ethanol levels in BM showed a good correlation with both cardiac and peripheral blood (*r *= 0.96 and *r *= 0.98, respectively; *P *< .0001). BM concentrations were generally slightly lower than peripheral blood, with BM-to-peripheral blood ratio of 0.87 ± 0.14 (range 0.63–0.99), while the cardiac blood-to-BM ratio averaged 1.03 ± 0.15 (range 0.74–1.39) [14].

Moreover, Winek and Jones investigated the relationship between ethanol concentrations in blood and rib BM in 18 human cadavers, reporting an average postmortem blood-to-BM ratio of 2.16 ± 0.32 (range 1.65–2.94). They suggested that the variability in this ratio was largely due to the lipid content of BM, as ethanol preferentially distributes into aqueous rather than lipid-rich compartments [13]. A subsequent and more extensive study by Winek and Esposito, 42 post-mortem cases were analyzed and their results confirmed these findings. Blood-to-rib BM ratios were consistent with the earlier study, averaging 1.94 ± 0.42 (range 1.34–3.22). After correcting BM ethanol concentrations for the aqueous fraction, the ratio narrowed to 1.26 ± 0.14 (range 0.97–1.66), and the correlation with blood ethanol levels improved markedly (*r *= 0.92–0.93; *P* not declared) [30]. Postmortem ethanol concentrations in BM and blood are summarized in Table 2.

**Table 2:** Overview of studies (1980–2019) reporting ethanol concentration in BM and its comparison with blood: number of cases, type of samples, blood and BM concentrations, mean ratio ± standard deviation (SD), correlation

References	Number of cases	Type of blood	Type of BM	Blood concentration mg/mL	BM concentration mg/g	Mean ratio ± SD	Correlation
Iskierka *et al*. [19]	59	Femoral blood	BM from iliac crest	0.68 ± 1.09	0.59 ± 0.96	–**	*r *= 0.97
Maeda *et al*. [14]	20	Cardiac blood	BM from thoracic vertebrae	–**	–**	Blood/BM: 1.03 ± 0.15	*r *= 0.96
		Peripheral blood		–**	–**	BM/Blood: 0.87 ± 0.14	*r *= 0.98
Winek and Jones [13]	18	Heart blood	BM from ribs	1.17–4.09	0.43–2.04	Blood/BM: 2.16 ± 0.32	–**
Winek and Esposito [30]	42	Heart blood	BM from ribs	0.03–0.39	0.02–1.75	Blood/BM: 1.94 ± 0.42 Blood/BM: 1.26 ± 0.14*	*r *= 0.92–0.93

* Ratio corrected for the aqueous fraction.

** Data not available.

Regarding ethyl glucuronide (EtG), the major non-oxidative metabolite of ethanol, Schloegl *et al*. studied its concentrations in rib BM to determine ante-mortem alcohol consumption [32]. BM EtG concentrations ranged from 0.77 to 9.36 μg/g and well correlated with blood alcohol concentrations (BAC 0.04–0.37 g%) (Table 1). The study suggested that rib BM, easily accessible during autopsies, is suitable for detecting postmortem alcohol biomarkers.

## Drugs stability in bone marrow

Drug stability in real samples is a critical issue in forensic toxicology and can be assessed at two levels: (i) pre-sampling stability, concerning the preservation of the body, and (ii) post-sampling stability, involving storage and laboratory handling of the samples.

As reported before, BM appears to be relatively well preserved prior to sampling due to its anatomical encapsulation, which protects it from microbial and environmental contamination. As a result, BM seems to retain xenobiotics for longer periods than soft tissues and fluids, which are more exposed to rapid decomposition [11]. Evidence from the literature supports its remarkable ability to preserve drugs even over prolonged postmortem intervals, highlighting its reliability as a matrix for toxicological analysis, as already reported in some studies mentioned in paragraph Illicit and therapeutic drugs in human bone marrow.

For example, Nagata *et al*. reported that amphetamine and methamphetamine remained detectable in BM samples from bones left outdoors for up to two years, without significant signs of degradation [31]. Triazolam was detected in BM from two individuals buried for 4 years and nearly identical BM concentrations of this BDZ were observed (0.36 and 0.37 ng/g). These findings further supported the hypothesis of ante-mortem sedation [26].

As mentioned before, Maeda *et al*. detected bromisovalum in BM collected from the two femora of a skeletonized body recovered on a forested slope seven months postmortem, at concentrations of 36.4 and 139.7 µg/g, despite advanced decomposition [25].

Further supporting evidence comes from a study investigating homicidal poisonings in a nursing home. Among 13 exhumed elderly victims, BM was the only available matrix in several cases. In two individuals, exhumed 30 months after death, cyamemazine was detected in BM at concentrations ranging from 229 to 681 ng/g, confirming drug administration prior to death [29].

Experimental studies in animal models provide additional insight. One study demonstrated that diazepam remained detectable in BM for longer periods than in blood or soft tissues. Although the sensitivity of detection decreased as the dose-to-death interval (DDI) increased, BM retained detectable levels longer than other matrices. This suggests that while decomposition influences detectability, BM offers superior preservation [33]^.^ Similarly, a second study examining fentanyl concentrations confirmed the persistence of the drug in BM even during advanced stages of decomposition. However, the authors acknowledged that stability is not absolute, and putrefaction may eventually alter analyte concentrations [34].

Collectively, these findings reinforce the notion that BM can serve as a long-term xenobiotic reservoir, often exceeding blood and other soft tissues in postmortem detectability.

Once BM is removed from its protective site, post-collection ­stability becomes critical. The stability of xenobiotics after sampling depends largely on storage temperature, duration, and ­handling. However, only limited research has addressed this issue, and just one study has focused on post-collection stability in human BM.

In humans, Winek *et al*. [35], assessed ethanol stability in BM samples stored at −6°C in 4 mL glass vials containing 3% sodium fluoride for 60 days. Despite the low-temperature storage and the presence of preservatives, all samples showed a decrease in ethanol concentrations, with losses averaging 25.9% (range 10.9–41%). These findings demonstrate that, even under controlled freezing conditions, ethanol is not stable in BM over time, and early analysis remains crucial for the accurate determination of ethanol levels in this matrix.

Animal studies provide additional insight into the behavior of xenobiotics stability in BM during its storage. In a controlled rabbit model, morphine and 6-AM were quantified in fresh BM and compared with samples stored for two months at room temperature. The results showed a 71% decrease in 6-AM concentrations and a complete degradation of morphine, demonstrating that opiate-derived analytes are particularly unstable in unfrozen BM [36].

Further evidence comes from an experimental study investigating postmortem ethanol formation in BM on 90 New Zealand white rabbits carcasses under different storage conditions. Carcasses were kept either at room temperature (19°C) or under refrigeration (3.5°C) for up to 28 days. In control animals not exposed to ethanol, BM stored at room temperature exhibited clear postmortem ethanol production due to microbial activity, with concentrations peaking around day 7 (average 78.3 mg %) and declining by day 21. On the other hand, refrigerated carcasses showed minimal or delayed ethanol formation: no ethanol was detected during the first 14 days, and only low concentrations appeared on day 21 and 28. These results demonstrate that BM is relatively protected against postmortem ethanol production, and that refrigeration further inhibits microbial activity in this matrix.

These results demonstrate that cooling is a decisive factor in limiting microbial activity within BM, highlighting the importance of rapid, low-temperature storage to maintain BM stability [37].

## Effects of post-mortem redistribution and decomposition on bone marrow

Accurate interpretation of postmortem xenobiotic concentrations remains one of the most challenging aspects of forensic toxicology. Among the factors that significantly affect postmortem levels in conventional biological matrices, PMR and decomposition are crucial. Although these phenomena arise from distinct physiological and biochemical mechanisms, they often overlap, modifying the distribution of substances across different body compartments and complicating the reconstruction of ante-mortem exposure.

PMR is an early postmortem process that begins shortly after death, involving the passive diffusion of drugs from highly perfused organs into the blood and surrounding soft tissues. The extent of PMR is influenced by the physicochemical properties of substances (e.g. lipophilicity, volume of distribution, and pKa) and by local postmortem conditions such as pH, temperature, and the degree of autolysis, which can lead to unpredictable changes in drug concentrations, particularly in central blood [38]. Decomposition begins immediately after death, with bacterial and fungal proliferation and acid-mediated tissue breakdown accelerating xenobiotic degradation and amplifying PMR. As decomposition progresses, blood and soft tissues lose structural integrity, become biochemically unstable, and highly susceptible to secondary alterations. These processes reduce the reliability of these matrices for postmortem analysis and complicate accurate determination of ante-mortem drug exposure [39].

The anatomical isolation of BM reduces its exposure to conditions that promote PMR, making it significantly more stable than blood, vitreous humor and soft tissues [29].

Experimental and observational studies consistently support the protective nature of BM. During progressive decomposition, xenobiotic concentrations in marrow remain more stable compared to other matrices. For instance, Watterson and Donohue [40] analyzed ketamine and norketamine levels in rat skeletal tissues across successive postmortem intervals and observed that drug concentrations were more stable in BM than in other tissues or blood as decomposition progressed. Furthermore, parent drug-to-metabolite ratios in BM tend to remain more consistent than in blood, providing a more accurate reflection of ante-mortem drug levels.

Histological investigations further support the resistance of BM to decomposition. Tattoli *et al*. evaluated postmortem BM morphology and found that, although cellularity declines over time (manifested by features such as vacuolar degeneration and granulocyte necrosis), the trabecular architecture of BM remains largely intact even after several weeks. Precursor cells of various hematopoietic lineages could still be identified, highlighting the gradual nature of BM autolysis [41]. Similarly, Roll *et al*. assessed the histopathology of 225 BM samples collected 12 to 140 hours postmortem, noting minimal autolytic changes in BM compared to severe degradation in other tissues from the same individuals [42].

## Conclusion

The reviewed literature consistently demonstrates the potential of BM as a valuable alternative matrix for postmortem toxicological investigations. BM relevance arises primarily from its anatomical encapsulation within the medullary cavity, where cortical bone forms an effective barrier against environmental exposure, microbial invasion, and fluid exchange. This protection reduced the susceptibility of this matrix to postmortem redistribution and putrefactive processes, and allowed BM to maintain relatively stable xenobiotic concentrations, also after they become undetectable in conventional matrices like blood or soft tissues. Across the studies, BM-to-blood exogenous concentration ratios vary systematically according to their physiochemical and pharmacokinetic properties. Lipophilic compounds tend to accumulate in BM, often reaching concentrations higher than those in blood, whereas hydrophilic substances typically display the opposite distribution. These trends highlight the importance of considering both exogenous-specific characteristics. Moreover, BM sampling sites are pivotal when interpreting BM toxicological results. Notably, rib BM showed stronger correlations with blood concentrations than femoral BM, likely reflating differences in red-to-yellow marrow composition and vascularity.

Collectively, these findings suggest that BM could be a matrix capable of preserving xenobiotics under conditions in which conventional specimens are often degraded, unavailable, or affected by postmortem redistribution. Nevertheless, further systematic research is needed to refine BM-blood correlation models, particularly by using standardized protocols for sampling and characterizing the substance-specific distribution.

## Data Availability

The data underlying this review article are available in the cited articles.
